# Midwives Registration—A General Practitioner's Protest

**Published:** 1891-03-14

**Authors:** W. D. Haslam


### Midwives' Registration—A General Practitioner's Protest.

By W. D. Haslam, M.D.

There is a mistaken idea abroad that general practitioners are opposed to the Midwives Bill from "sordid interests," because the Bill would, if passed in its present form, create a lower grade of obstetric practitioners.

Whatever may be the opinion of general practitioners regarding this Bill, a certain amount of deference is due to it, because they are in a much better position to determine how far the demand for midwives by the public is justified than are the promoters of this Bill, who never come into contact with the poor except in hospital practice. It is the rank and file of the profession who know best how the poor live, and what their requirements may be, for they visit them in their homes.

Dr. Aveling says "the great mass of the profession is in favour of the Bill." This is a bold statement to make when it has never been canvassed, and in face of such direct opposition as has been manifested in the medical journals. Nevertheless, the statement contains at least a germ of the truth, viz., that the great mass of the profession are in favour of the principle that midwives in the future shall be better educated in their duties than they have been in the past. But here I venture to say, so far as this Bill is concerned, their support ends, for they cannot admit the necessity of creating midwives in numbers out of all proportion to the demand, at the same time conferring upon them increased functions and freedom, with legal protection without adequate control.

The laity, including some of the M.P.'s who are in support of this Bill, are under the impression that there is urgent need for skilled midwives because they have heard of instances in which families have suffered in consequence of the ignorance of so-called midwives. No doubt such cases do occur, but they are infrequent, because the present midwife is so dependent upon local practitioners in times of difficulty or danger. But in this very helplessness there is an element of safety to the patient, which will be swept away if midwives are to be made legally independent. The laity also firmly believe that the Midwives Bill is to benefit the poor, for they think that no one would employ a midwife unless driven to do so through poverty. Every-day experience does not confirm this belief, and it must be borne in mind that there is a community in every town not absolutely poor, but whose livelihood may be precarious, and who, for various reasons, would employ the midwife rather than have their names entered in books, because they prefer to live unknown, besides cases of illegitimacy and abortion, which, unfortunately, sometimes occur in the best-regulated families. Do people ever consider the vast machinery which is already at work to give free professional attendance to the poor at their own homes, in hospitals, infirmaries, and havens?

It is too easy in these days to throw would-be philanthropists into a state of hysterical activity, and not until the fit is over do they become acquainted with essential details.

If, however, the Bill is projected for the sake of the deserving poor, it should be placed under the administration of the poor law authorities; for in every district, urban or rural, they have the best means at their disposal for regulating the number of midwives required, the people that need their services, and there would also be at hand the district medical officer to assist in abnormal cases.

The Bill as it stands invites "women to be examined who are desirous of becoming midwives," also "to grant to every woman who passes," &c., &c.

It is prepared to flood the country with an unlimited supply when the demand for that supply is very limited. The result of an over supply would mean deficient work to some midwives and privation to others; there would follow the temptation to nefarious practices; and there would be various degrees from the best type of midwife right down to the female abortionist. It is a better quality of midwife that is wanted, not a larger quantity; not increased functions, but controlled action; amendment, not a new Bill.

It has never been explained why the present system of training has failed. For years past the lying-in hospitals and the Obstetrical Society of London have educated and examined women, and the latter has afterwards given certificates of proficiency to those deemed worthy of holding such certificates. Now who is to blame if the certificated midwife of to-day is incompetent after having been examined? It is a matter of importance that this question should be sifted, or the same mistakes will be propagated in the future. The same men who have been responsible for this certification in the past will expect similar appointments in the future. But until the cause has been discovered the remedy will not be forthcoming.

The women who practice as midwives now without any kind of training whatever ought to be suppressed in common with other irregular practitioners, and the new Bill has this in view.

There is one more point I would like to allude to. It has been the custom of practitioners to attend no patient in labour unless a previous engagement has been made. Will general practitioners care to be roused up at all times to get a midwife out of trouble? I fancy not. This is only another reason why midwives should, after their registration, act in conjunction with the poor law authorities in every district.

Without desiring to offer any opposition to the main principle of the Bill, I sincerely hope that whatever Bill becomes law will be found to be workable, in harmony with general practitioners and with the public need.

